# Effects of Different Pre-Cooling Methods on the Shelf Life and Quality of Sweet Corn (*Zea mays* L.)

**DOI:** 10.3390/plants12122370

**Published:** 2023-06-19

**Authors:** Chi Zhang, Pengcheng Zhou, Jun Mei, Jing Xie

**Affiliations:** 1 College of Food Science and Technology, Shanghai Ocean University, Shanghai 201306, China; m210300872@st.shou.edu.cn (C.Z.); m190300698@st.shou.edu.cn (P.Z.); 2 Key Laboratory of Aquatic Products High Quality Utilization, Storage and Transportation (Co-Construction by Ministry and Province), Ministry of Agriculture and Rural Affairs, Shanghai 201306, China; 3 National Experimental Teaching Demonstration Center for Food Science and Engineering, Shanghai Ocean University, Shanghai 201306, China; 4 Shanghai Engineering Research Center of Aquatic Product Processing and Preservation, Shanghai 201306, China; 5 Shanghai Professional Technology Service Platform on Cold Chain Equipment Performance and Energy Saving Evaluation, Shanghai 201306, China

**Keywords:** sweet corn, pre-cooling methods, cold storage, shelf life

## Abstract

The strong wind pre-cooling (SWPC), ice water pre-cooling (IWPC), vacuum pre-cooling (VPC), natural convection pre-cooling (NCPC), and slurry ice pre-cooling (SIPC) techniques were used to pre-cool the fresh sweet corn (*Zea mays* L.), and then the pre-cooling treated sweet corn samples were stored at 4 °C for 28 days. During refrigeration, quality indicators, such as hardness, water loss, color, soluble solids content, and soluble sugar, were determined. In addition, oxidation indicators, such as peroxidase, catalase, ascorbic acid-peroxidase activity, and carotene content, were also measured. The results showed that the main problems of sweet corn during cold storage were water loss and respiration. The pre-cooling speed of SWPC is the fastest, and the latent heat of sweet corn can be removed in only 31 min. SWPC and IWPC could reduce the loss of fruit quality, maintain good color and hardness, inhibit the decrease of water, soluble solids, soluble sugars, and carotenoid contents, maintain balance between POD, APX, and CAT, and extend the shelf life of sweet corn. The shelf life of SWPC and IWPC corn reached 28 days, 14 days longer than SIPC and VPC treated samples, and 7 days longer than NCPC treated samples. Therefore, SWPC and IWPC are the appropriate methods to pre-cool the sweet corn before cold storage.

## 1. Introduction

Sweet corn (*Zea mays* L.) is among the most important cereals grown worldwide; sweet corn is picked just before maturity, a stage when corn contains a lot of carbohydrates and water [1]. Therefore, fresh sweet corn is susceptible to spoilage. After harvest, sugar and moisture loss, fungal infection, and enzymatic browning are the main problems affecting the quality of sweet corn, causing a loss after harvest [2]. Pre-cooling is an important treatment to slow down the biological processes to maintain the quality [3]. It is thought to quickly remove in-field heat from newly picked sweet corn, so that the sweet corn temperature can fall rapidly from the field temperature to the proper temperature [4].

In recent years, technologies commonly used for pre-cooling of fruits and vegetables consist of strong wind pre-cooling (SWPC), ice water pre-cooling (IWPC), vacuum pre-cooling (VPC), and natural convection pre-cooling (NCPC) [3]. Wang et al. found that SWPC improves the air delivery rate, and extends the storage time of iceberg lettuces [5]. Post-harvest pre-cooling of fruits and vegetables can also delay color deterioration [6]. IWPC is a fast, simple, and cost-effective method of cooling. Water has high heat conductivity, the temperature falls rapidly due to the full contact of ice water and product surface [7]. IWPC could protect the structure of the cell wall of rock melon and keep its mass during the storage process [8]. IWPC is also capable of retaining moisture in the fruit and vegetable products [9]. IWPC can also mitigate the decline in parsley fresh weight by reducing the consumption of solids such as carbohydrates [10]. However, IWPC is not appropriate to all fruits and vegetables, as the water content of some fruits and vegetables is inversely proportional to their mass [11]. VPC can improve the activity of antioxidant enzymes and increase the scavenging rate of free radicals [12]. VPC enables rapid vaporization of water to release latent heat; however, it is not suitable for pre-cooling the larger fruits and vegetables, such as apple, sweet potato, etc. [13]. Zhu et al. showed that the temperature and vacuum tolerances variation of the petioles and leaves of P. alba was differential when pre-cooled, and the leaf paraxial epidermis was more sensitive to Vc and the internal water gradient caused more serious cell damage [14]. Bellas et al. found that the cooling ability of ice slurry was 5–6 times greater than that of regular chilled water [15].

The choice of pre-cooling method is dependent on the product’s characteristics, transportation terms, and costs [16]. Different products have different acceptance of different pre-cooling methods, and some products may be damaged by certain pre-cooling techniques [17]. In the present study, different pre-cooling methods, including VPC, SIPC, IWPC, SWPC, and NCPC, were evaluated in terms of sensory characteristics, water loss, hardness, color, the total soluble solids, soluble sugar, carotenoids, ascorbate-peroxidase, catalase, and peroxidase.

## 2. Results and Discussion

### 2.1. Pre-Cooling Curve

**Figure 1:**
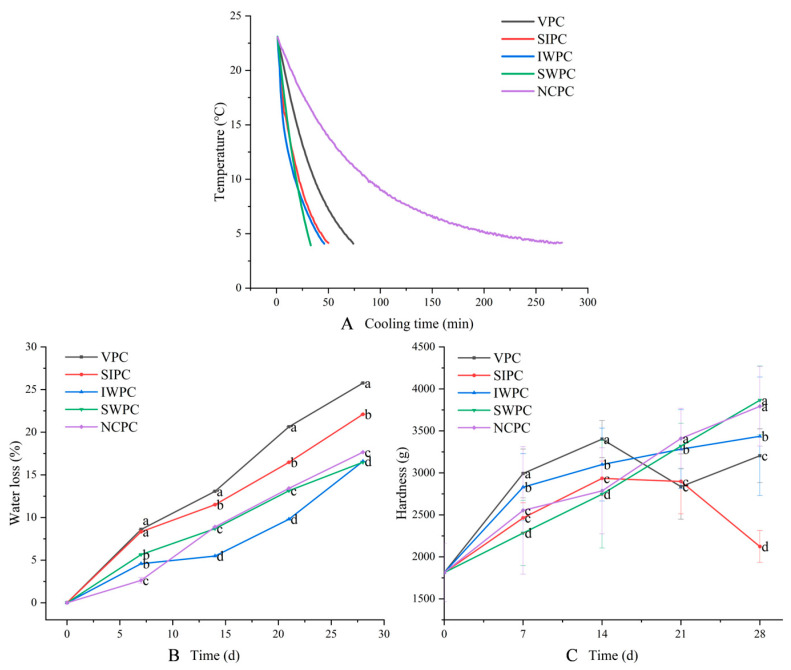
Analysis of (**A**) pre-cooling curve, (**B**) water loss, and (**C**) hardness. (**A**) indicates the cooling time of sweet corn under the effect of different pre-cooling methods. (**B**) shows the water loss of sweet corn under the effect of different pre-cooling methods. (**C**) shows the change in hardness of sweet corn under the effect of different pre-cooling methods. Different colors in (**A**–**C**) indicate different pre-cooling methods (Black-VPC, Red-SIPC, Blue-IWPC, Green-SWPC, Violet-NCPC). Different lowercase letters indicate significant difference (*p* < 0.05).

Fruits and vegetables are pre-cooled immediately after harvest, and the outlook, taste, and nutrition are better with cold storage [18]. SWPC has a fast cooling speed, low cost, and causes little damage to horticultural products [19]. As shown in Figure 1A, all samples started with a uniform temperature of 23 °C. The SWPC and IWPC samples were able to pre-cool at a faster rate and therefore reached the desired temperature in less time than the other samples. This resulted in a faster release of latent heat from sweet corn, which inhibited respiration [20] and transpiration [21], and thus maintained better storage quality. On the contrary, the NCPC treatment had the longest pre-cooling time and could not release latent heat quickly. This problem was also present in the VPC samples, probably due to the vacuum pressure not being high enough; however, if the pressure was increased further, the quality of the sweet corn would deteriorate further. The influence of different rates of cooling methods on the mass of enoki mushrooms was studied by Mittal et al. Their study claimed that VPC maintained the best physicochemical and organoleptic quality, followed by SWPC and NCPC [22]. Despite the short cooling time of IWPC, it still caused the deterioration of the mushrooms due to the high moisture content [23]. This was not entirely consistent with our experimental results, and the possible reason for this was that button mushrooms belonged to the fungus category and sweet corn belonged to the vegetable category. They reported the effect of cooling rate on the quality of produce is related to the product type [16].

### 2.2. Water Loss

The research showed that the average water content of corn is 53.4%. The transpiration and respiration during cold storage need a lot of water, which increases the sweet corn water loss rate [24]. The water loss rate concomitantly increased with the storage time (Figure 1B). Water loss is a very significant physiological feature of sweet corn, as it has a direct impact on appearance and taste [25]. The results showed that IWPC treatment could delay the decline of water loss rate during storage as the IWPC treatment made sweet corn absorb a lot of water, thereby reducing its own water consumption and evaporation. The sweet corns became shriveled on the 21st day and lose their market values due to the excessive loss of water. The pre-cooling treatments, except IWPC, increased the water loss of sweet corn during cold storage as the pre-cooling treatment sped up the water transpiration on the surface [26]. The quality of VPC-treated sweet corn was lower than that of other samples because of the large amount of water loss [27]. To undergo vacuum cooling. The product must have a porous structure to release the evaporated water. In addition, VPC treatment can reduce the temperature by evaporation of water, which leads to significant changes in cell membrane and cell wall space, triggering changes in cell morphology and destruction of cell structure, increasing the membrane permeability [28]. Zhu et al. found that VPC-treated lettuce had severe water loss [29]. Therefore, the products should be pre-wetted prior to pre-cooling, thus reducing the water loss of the products, which is consistent with the results obtained by our IWPC samples. However, sweet corn is not suitable for VPC due to its lack of porous structure.

### 2.3. Hardness

Hardness can reflect the maturity and aging degree of corn during storage [30], and it is likewise a major classification criterion for sweet corn [31]. At the early stage of storage, the hardness increased quickly, indicating that sweet corn was rapidly maturing (Figure 1C). The hardness of IWPC samples increased slowest in the middle and late stages of storage because IWPC treatment could maintain higher humidity. This could inhibit metabolic processes, such as respiration, in sweet corn, reducing nutrient consumption and maintaining tenderness. The hardness results of IWPC, NCPC, and SWPC were similar with the water loss results. The hardness of the SIPC and VPC samples decreased at the later stages of storage, probably due to the most serious water loss in these two treated samples and the cell wall degradation led to a greater degree of corn kernel collapse, which affected the hardness. Loss of hardness caused by cell wall and starch degradation may result in the physical damages during transport and susceptibility of sweet corn to pathogens. Therefore, the hardness loss was related to the loss in other aspects [32]. The actual hardness changes of apple [33] and Pipa [34] were consistent with the results of IWPC, SWPC, and NCPC samples in the current experiments.

### 2.4. Color

Color is a product feature that is valued by consumers because it visually indicates freshness [13]. The L* and b* are used to reflect the freshness of sweet corn [35]. The greater the positive L* and b* value, the more the color tends to be brighter and yellow. The L* and b* values of IWPC samples were greater than those of other samples during storage (Table 1), which indicated that IWPC samples could better maintain the brightness of sweet corn. The IWPC treatment was able to maintain a high moisture content and soluble solids content in sweet corn. Except for the IWPC samples on the 7th day, the L* value decreased slowly throughout the storage period, indicating that the brightness of sweet corn gradually decreased with the extension of storage time, which was caused by the gradual water loss of sweet corn during storage. This was similar with the water loss results. During storage, although there were ups and downs, the b* of all treatment groups showed an overall decreasing trend. The b* of the IWPC group were lower than those of the SIPC group only at day 14, and the b* of IWPC were the highest at all other time points, indicating that IWPC could effectively maintain the yellowness of sweet corn. The results showed that IWPC treatment could better maintain the freshness and yellowness of sweet corn, thus improving its commercial value. He et al. found that the change of L* value of mushrooms in the pre-cooling process was consistent with our experimental results [36].

### 2.5. Total Soluble Solids (TSS)

**Figure 2:**
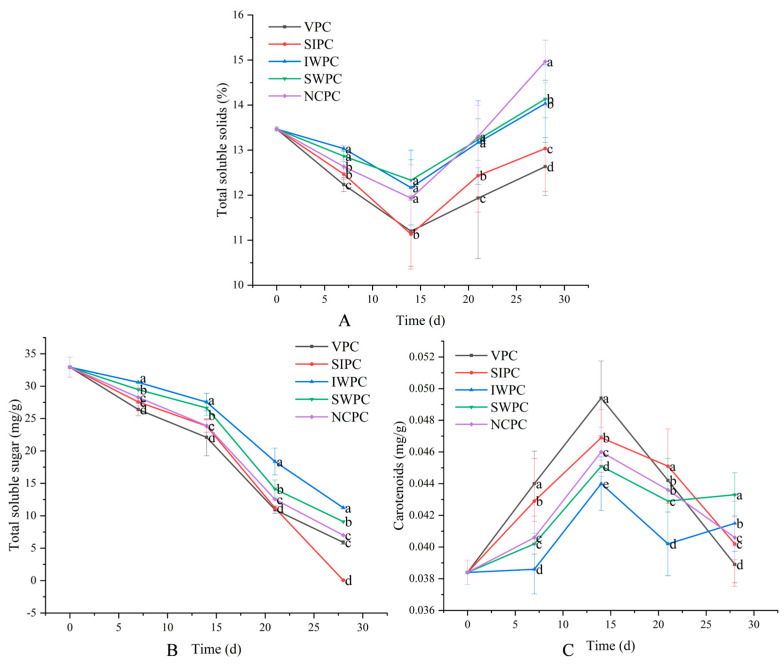
Analysis of (**A**) solid substances, (**B**) total soluble sugar, and (**C**) carotenoids. (**A**) indicates the solid substances of sweet corn under the effect of different pre-cooling methods. (**B**) shows the total soluble sugar of sweet corn under the effect of different pre-cooling methods. (**C**) shows the change in carotenoids of sweet corn under the effect of different pre-cooling methods. Different colors in (**A**–**C**) indicate different pre-cooling methods (Black-VPC, Red-SIPC, Blue-IWPC, Green-SWPC, Violet-NCPC). Different lowercase letters indicate significant difference (*p* < 0.05).

TSS is an important index to judge the storage effect of sweet corn [37]. The TSS contents of all samples decreased first and then increased during storage (Figure 2A). Respiration resulted in a decrease in TSS content [38]. The respiratory metabolic activity was vigorous, and its own nutrients were consumed in large quantities, leading to the decrease of TSS contents [39]. The TSS contents of VPC samples were always lower than that of other samples, which due to the VPC treatment disrupting the tissue structure of sweet corn and the samples treated with VPC lost the most water, resulting in more soluble solids flowing away with the water than the other samples. The ash and fat contents generally increased with the extension of storage time [40], while the fiber, protein, and carbohydrate contents decreased [41]. The reason for the gradual increase in TSS at the later stage of storage was that ash and fat contents increased faster than protein and carbohydrate consumption [42]. The TSS contents of all samples reached the highest in the late harvest period, mainly concentrated at about 28 days. There was no correlation between results at later stages of TSS storage and total sugar, suggesting that other components, such as organic acids, vitamins, phenolic compounds, pectin, starch, and protein, may influence this variable. Marrufo-Díaz et al. also came to a similar conclusion; they found that soluble solids content was not positively correlated with changes in total soluble sugars [43]. In general, SWPC and IWPC samples could effectively delay the decline of TSS contents compared with other samples because both treatments were effective in reducing respiration and transpiration of sweet corn and had lower water loss than the other samples. Han et al. and Leccese et al. found that mulberry fruit [44] and apricot [45] could maintain good TSS contents in the early stage of storage after pre-cooling treatment, which was consistent with our experimental results.

### 2.6. Soluble Sugar

Soluble sugar is an important index of taste quality of sweet corn, which directly determines the sweetness of fresh corn [46]. High sugar content makes sweet corn more vulnerable to microbial damage, which affects the eating quality of fresh fruit ears [47]. The contents of soluble sugar contents decreased gradually during storage (Figure 2B) as the metabolic rate of fresh corn was high [48], and the sucrose supply transported by leaves was no longer obtained after the ear harvested in vitro. The respiration played a dominant role, and the contents of sucrose and soluble sugar in sweet corn gradually decreased after harvest. The IWPC treatment could well delay the decrease of soluble sugar content, and the effects of VPC and SIPC treatments were poor. The VPC and SIPC treatments destroyed the corn tissue structure, and the VPC and SIPC samples lost water more severely, resulting in the loss of soluble sugars more easily than the other samples. However, the IWPC protected the corn tissue structure and delayed the water loss of sweet corn. Ding et al. found that IWPC retarded the decline of soluble sugars in sweet corn [49]. Xu et al. also showed that IWPC could reduce the loss of soluble sugars better than other pre-cooling methods [50].

### 2.7. Carotenoids

ROS are generated during normal metabolic processes, such as fruit development and ripening, acting as both signaling molecules responsible for tuning cellular biological functions (i.e., promoting cellular proliferation and differentiation) but also as toxic by-products generated during aerobic metabolism [51]. As antioxidants, carotenoids are capable of inactivating ROS and may therefore help delay or prevent oxidative damage [52]. Lipoxygenase, peroxidase, and other enzymes can also participate in the oxidation of carotenoids [53]. The carotenoids of all samples increased in the early stage of storage (Figure 2C), resulting from the strong respiration [54]. At the later stage of storage, the oxidative degradation rate of carotenoids exceeded the water loss rate of sweet corn, resulting in the decrease of carotenoid contents. There was no significant change in carotenoid content in IWPC samples, which may be due to higher water content and lower respiratory efficiency. This is also reflected in the results of water loss, TSS, and soluble sugar results. VPC had a poor effect on the retention of carotenoids in sweet corns and the oxidation rate of carotenoids was faster. Zainal et al. [55] found that the carotenoid contents of water-cooled melons were well maintained, which was consistent with the experimental results of the IWPC and SWPC samples.

### 2.8. APX

**Figure 3:**
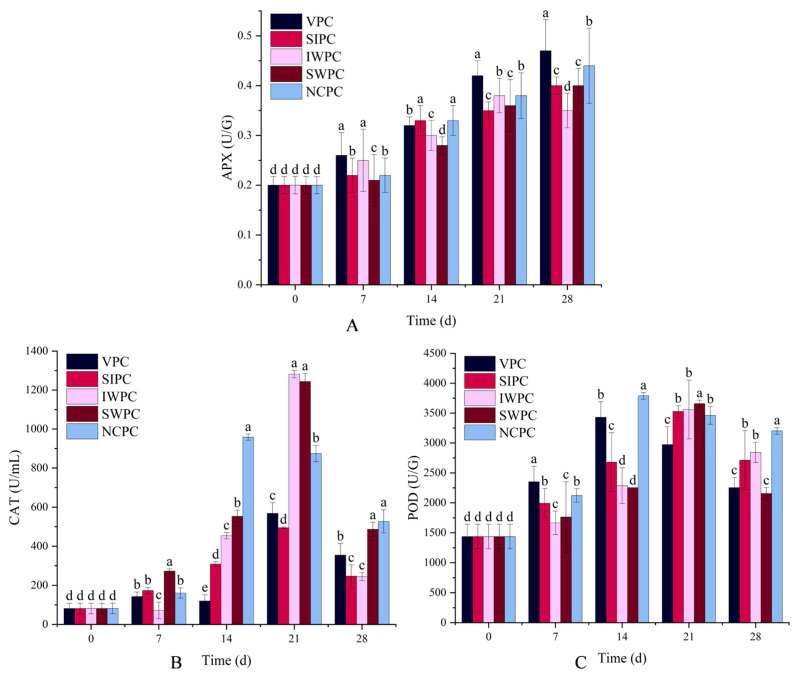
Analysis of (**A**) APX, (**B**) CAT, and (**C**) POD. (**A**) indicates the APX of sweet corn under the effect of different pre-cooling methods. (**B**) shows the CAT of sweet corn under the effect of different pre-cooling methods. (**C**) shows the change in POD of sweet corn under the effect of different pre-cooling methods. Different colors in (**A**–**C**) indicate different pre-cooling methods (Black-VPC, Red-SIPC, Pink-IWPC, Brown-SWPC, Blue-NCPC). Different lowercase letters indicate significant difference (*p* < 0.05).

In the process of storage, sweet corn is prone to oxidation due to its rich nutrients and environmental factors. Reactive oxygen species (ROS) is beneficial to improve the disease resistance of plants, but excessive ROS can affect DNA replication and protein synthesis, leading to fruit senescence [56]. APX is an important antioxidant enzyme in cells, which can remove excessive ROS in sweet corn tissues and maintain high antioxidant performance, thereby protecting membrane structure and inhibiting oxidation process [26]. The APX enzyme activity of IWPC samples increased at the beginning and then decreased (Figure 3A), and other samples showed a gradual upward trend. The pre-cooling treatment effectively increased the APX, enhancing the antioxidant capacity of the cells and regulating the ROS metabolism. Under the stress of external force, the metabolic balance of reactive oxygen species was broken, leading to the accumulation of free radicals and the aggravation of membrane lipid peroxidation, which induced the corn senescence [57]. The VPC could effectively increase APX, but the oxidation loss of sweet corn could not be reduced well, indicating that the increase of APX by VPC treatment was not enough to alleviate the oxidation of sweet corn. The relationship between APX and the degree of oxidation showed that the quality of pre-cooling sweet corn was the result of the combined effect of many factors, and the change of individual indicators could not prove the inapplicability of a certain pre-cooling method. The enhancement of antioxidant enzyme ability may be related to the up-regulation of antioxidant gene expression after pre-cooling treatment [58]. For mango fruits, pre-cooling treatment significantly increased the activity of APX [59], which was consistent with the current results.

### 2.9. CAT

OH^−^ is produced by the oxidation reaction of sweet corn during storage, and CAT could delay the oxidation reaction [60]. Previous studies demonstrated that hydrogen peroxide (H_2_O_2_), the most stable type of ROS, played a vital role in the ripening and senescence of various fruit [61]. At high concentration, H_2_O_2_ can oxidize cysteine (-SH) and inhibit enzyme activity [62], and eventually resulted in sweet corn senescence. CAT specifically converts H_2_O_2_ into H_2_O and O_2_ [63]. CAT in all samples increased in the early storage (Figure 3B), indicating that pre-cooling could effectively maintain the stability of free radical metabolism of sweet corn. The slow growth of CAT at the early stage of storage may be due to the high freshness and low oxidation degree of sweet corn. With the extension of storage time, the oxidation degree of sweet corn was deepened, and CAT increased, which inhibited the oxidation process. The decrease in CAT activity in late storage could be explained by the rapid increase in the H_2_O_2_ content of sweet corn in late storage [64]. As can be seen from Figure 3B, the CAT activities of IWPC and SWPC samples were significantly higher than that of other samples in the later stage (*p* < 0.05), indicating that IWPC and SWPC could better reduce the damage to macromolecules by free radicals [65]. This result was confirmed in the TSS and soluble sugar results. These pre-cooling treatments delayed the physiological and biochemical reactions of sweet corn, delayed the senescence, and improved the quality, which was confirmed by the sensory evaluation results. Zhu et al. also reported that pre-cooling treatment could increase CAT in the early storage period [17].

### 2.10. POD

POD can cooperate with the protective enzyme system to effectively remove free radicals [58], which is closely related to the antioxidant capacity of plants. It is a defense line in the antioxidant system of sweet corn and changes O_2_ disproportionately to H_2_O_2_ [66]. The POD activity of sweet corn treated by different pre-cooling methods showed an upward trend at the beginning (Figure 3C), which improved the ability of sweet corn to resist pathogens. IWPC, SWPC, and SIPC samples were able to delay the arrival of POD peak, and the delay of POD peak meant the delay of senescence. The above results showed that IWPC and SWPC were effective in preventing the oxidation of sweet corn, which was consistent with the results obtained in TSS, soluble sugar and sensory evaluation results. Xiao et al. showed that the increase in POD content tended to represent a weakening of oxidation [67]. Li et al. found that pre-cooling increased the POD activity of mangoes [26]. The balance between antioxidant enzymes is important to determine the relative stability between H_2_O_2_ and ROS.

### 2.11. Sensory Characteristics

**Figure 4:**
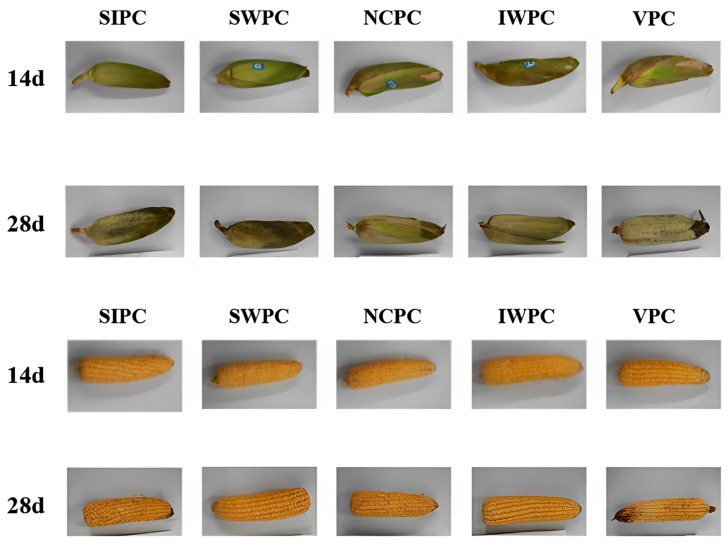
Sensory characteristics of sweet corn with different pre-cooling methods during storage.

Sensory characteristics are the key factors affecting consumers’ purchase intention and the most intuitive index to judge the freshness of sweet corn [68]. All the samples showed deterioration trends during storage (Figure 4). SWPC still showed good sensory quality on the 14th day, but the IWPC, NCPC, and VPC groups had significantly better quality. The reason for the mildew in IWPC and NCPC samples was the low rate of early water loss and high-water content, which made it easier for mold to grow. The VPC samples had no significant difference in grain quality and had a uniform color and full kernels on the 14th day. Liu et al. also found that pre-cooling could maintain good visual quality in the early stage of storage [69]. Other results in this experiment showed that the IWPC group had lower water loss at the end of storage (28 d) (Figure 1B), less variation in L* and b* (Table 1), and a higher soluble sugar content (Figure 2B). These indicators were closely related to sensory quality. Specifically, IWPC produced best appearance as compared to other samples through visual observation. This study showed that low temperature storage after pre-cooling treatment was an effective method to maintain the quality and prolong the shelf life of sweet corn. Specifically, IWPC produced the best appearance as compared to other samples through visual observation.

The deterioration of corn quality during storage was mainly carried out by water loss, color change, firmness change, and nutrient substance change. These changes were ultimately reflected in changes in the appearance of sweet corn. At the same time, another major cause of the decline in storage quality was the oxidation of sweet corn. In simple terms, the high production of H_2_O_2_ deactivated enzymes in sweet corn, which led to dysregulation of normal cellular processes, and ultimately led to senescence and quality loss of sweet corn. While POD could inhibit the formation of H_2_O_2_, CAT and APX could accelerate the degradation of H_2_O_2_. Under the action of these three enzymes, the senescence of sweet corn has been effectively alleviated. The results and analysis showed that SWPC and IWPC could better alleviate the senescence and quality deterioration of sweet corn and prolong its shelf life.

## 3. Materials and Methods

### 3.1. Sample Preparation

The variety of sweet corn was “Shenke Tian No. 2”. Sweet corn was picked from the fields of Fengxian District (121.581965° E, 30.9785° N), Shanghai, and shipped directly to the lab for processing. The sweet corn was simply selected to be similar in size (400 g), close in character, and undamaged.

### 3.2. Pre-Cooling Methods

The following five kinds of pre-cooling treatments were carried out:

(i) SWPC: the sweet corn was placed directly into trays and pre-cooled in a 0 °C freezer with under a powerful fan (DJ-12, Zhengfa Technology Co., Ltd., Kunming, China);

(ii) IWPC: the corn was pre-cooled directly in a container containing a mixture of 0 °C ice water;

(iii) VPC: the corn is placed in a vacuum cooling chamber (Vac-0.2, Shanghai Fresh Green Vacuum Preservation Equipment Co., Ltd., Shanghai, China) with the vacuum set at 600 Pa. Some water drops were placed on the surface of the corn to ensure heat was removed from the corn;

(iv) NCPC (Control): The corn was placed directly in a thermotank (BPS-100CL, Shanghai Yiheng Scientific Instrument Co., Ltd., Shanghai, China) at 4 °C;

(v) SIPC: The corn was buried in fluidized ice with a 3:1 ratio of ice to sweet corn.

**Figure 5:**
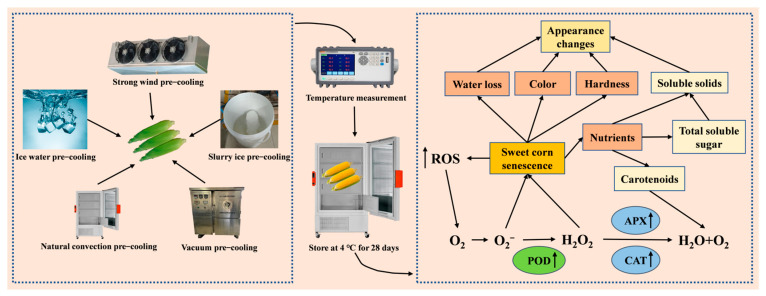
Experimental procedure and mechanism of sweet corn quality deterioration.

Thermocouple wires (JK-3040, JINKO, Shanghai, China) were inserted into the corncob to measure the temperature change of the sweet corn. When the temperature of the corn reached 4 °C, the sweet corn was transferred to a thermotank (BPS-100CL, Shanghai Yiheng Scientific Instrument Co., Ltd., Shanghai, China) at 4 °C. A flow chart of the experiments described in this paper is shown in Figure 5.

### 3.3. Determination of Sweet Corn Quality

#### 3.3.1. Sensory Characteristics

When sweet corn was stored to the 14th day and 28th day, sensory quality was judged by appearance, taste, and mildew odor. Ten trained assessors evaluate each sample. The criteria for assessment are listed in Table 2.

#### 3.3.2. The Weight Loss

The sweet corn was weighed before and after pre-cooling treatments and weight loss was calculated using the following equation:

(1) $$Weight\;loss=\frac{w_{initial}-w_{test}}{w_{initial}}\times 100\%$$

#### 3.3.3. Hardness

Hardness was measured by a texture analyzer (TA. XT Plus, Stable Micro Systems, UK). The experiment was conducted using the P6 probe of the texture analyzer with pre-test speed set to 1 mm/s, test speed set to 2 mm/s, strain set to 40%, and time set to 5 s [70].

#### 3.3.4. Color

The chroma meter (CR-400, Konica Minolta, Tokyo, Japan) was used to measure the color (L*, b*) of sweet corn. The sample was equilibrated to room temperature. The colorimeter was calibrated before measurement and the color was measured from three different random positions for each measurement [71].

#### 3.3.5. Total Soluble Solids

Sweet corn kernels were homogenized and centrifuged at 15,285× *g* at 4 °C for 20 min. The supernatants were collected and tested using a digital display refractometer (WYT-32, Quanzhou optical, Quanzhou, China). For each daily experiment, five sweet corns for each sample were used to determine the total soluble solids (TSS) content [72].

#### 3.3.6. Total Soluble Sugar

Experiments were performed according to the method of Brenes et al. [35]. Five grams of the ground sample was placed into a 50 mL tube and extracted twice with 25 mL of a mixture of ethanol and water (50:50, *v*/*v*) and centrifuged at 2250× *g* at 4 °C for 20 min. The supernatants were combined, and 2 mL of the supernatant was filtered through a 0.2 µm water syringe filter to measure the sugar content.

#### 3.3.7. Carotenoids

Carotenoids were determined according to the method of Deng et al. [73]. The samples were well ground in hexane: acetone (1:1, *v*/*v*) solution using a mortar and pestle and extracted on a shaker at 270 rpm for 1 h at room temperature. The supernatant was measured at 450 nm using a spectrophotometer.

#### 3.3.8. Ascorbate-Peroxidase (APX)

APX activity was measured with the Ascorbate Peroxidase (APX) Activity Assay Kit (BC0225, Beijing Solarbio Science & Technology Co., Ltd., Beijing, China).

#### 3.3.9. Catalase (CAT)

CAT activity was measured with the Catalase (CAT) Activity Assay Kit (BC0205, Beijing Solarbio Science & Technology Co., Ltd., Beijing, China).

#### 3.3.10. Peroxidase (POD)

POD activity was measured with the Peroxidase (POD) Activity Assay Kit (BC0095, Beijing Solarbio Science & Technology Co., Ltd., Beijing, China).

### 3.4. Statistical Analysis

All the experiments were repeated at least three times. Data were analyzed on SPSS 26.0 software using ANOVA with a Duncan’s test and expressed as mean ± standard deviation (SD). Data with *p* < 0.05 were considered to be significantly different. Origin 2021 was used for mapping.

## 4. Conclusions

The effects of different pre-cooling methods (SWPC, IWPC, VPC, NCPC, and SIPC) on the quality of sweet corn stored at 4 °C were studied. IWPC and SWPC samples had a shorter pre-cooling time. IWPC- and SWPC-treated sweet corn could effectively maintain good moisture content, color, and hardness during 28 days of storage at 4 ℃. The results showed that the IWPC and SWPC treatments could effectively inhibit the decline of TSS, soluble sugar, and carotenoid contents, and maintain the high CAT and POD enzyme activities. In contrast, VPC treatment led to water loss from sweet corn, which affected metabolism and antioxidant enzyme activities, leading to accelerated deterioration of sweet corn. Therefore, SWPC and IWPC treatments could effectively maintain the storage quality of sweet corn and have practical application value for prolonging the shelf life of sweet corn.

## Figures and Tables

**Table 1:** The color of sweet corn with different pre-cooling methods during storage.

Color	Storage Time	Pre-Cooling Methods				
	Day	VPC	SIPC	IWPC	SWPC	NCPC
L*	0	81.47 ± 0.03	81.47 ± 0.03	81.47 ± 0.03	81.47 ± 0.03	81.47 ± 0.03
	7	81.44 ± 1.27 a	81.24 ± 0.1 a	79.33 ± 0.81 b	82.12 ± 0.53 a	80.87 ± 0.75 a
	14	78.42 ± 0.07 b	78.1 ± 1.37 b	80.14 ± 0.1 a	77.28 ± 1.14 b	78.87 ± 0.61 a
	21	77.56 ± 1 b	75.48 ± 1.1 c	78.86 ± 0.59 a	76.96 ± 0.73 b	76.12 ± 0.63 c
	28	74.88 ± 0.8 b	75.29 ± 0.82 b	77.88 ± 0.49 a	75.55 ± 1.09 b	75.49 ± 0.23 b
b*	0	40.07 ± 0.03	40.07 ± 0.03	40.07 ± 0.03	40.07 ± 0.03	40.07 ± 0.03
	7	42.38 ± 0.78 b	39.79 ± 3.08 b	45.68 ± 3.27 a	31.64 ± 5.37 c	40.27 ± 3.18 b
	14	33.33 ± 0.23 b	38.44 ± 2.17 a	38.16 ± 0.59 a	36.68 ± 0.26 a	31.54 ± 0.99 b
	21	40.54 ± 2.03 a	35.85 ± 4.14 c	38.64 ± 2.14 b	37.99 ± 0.85 b	36.07 ± 3.63 c
	28	31.98 ± 1.15 c	36.6 ± 4.8 b	37.82 ± 2.95 a	35.95 ± 0.27 b	36.94 ± 6.64 b

Note: Different lowercase letters indicate the significance of different treatment groups.

**Table 2:** Sensory evaluation criteria of sweet corn.

Color and Shape	Odor and Mildew
Uniform color, full granules, and smooth skin	No mildew, impurities, odor
Uniform color, skin micro-folds	Almost no mildew and odor, slight impurities
Dark color, unsaturated granules, skin folds	Mildew, impurities, odor

## Data Availability

And on reasonable request, the corresponding author will provide the data used or analyzed during this investigation.
